# Specific Activation of the G Protein-coupled Receptor BNGR-A21 by the Neuropeptide Corazonin from the Silkworm, *Bombyx mori*, Dually Couples to the G_q_ and G_s_ Signaling Cascades*

**DOI:** 10.1074/jbc.M112.441675

**Published:** 2013-03-01

**Authors:** Jingwen Yang, Haishan Huang, Huipeng Yang, Xiaobai He, Xue Jiang, Ying Shi, Damirin Alatangaole, Liangen Shi, Naiming Zhou

**Affiliations:** From the ‡ Department of Economic Zoology, College of Animal Sciences, and; the ¶ Institute of Biochemistry, College of Life Sciences, Zijingang Campus, Zhejiang University, Hangzhou, Zhejiang 310058,; the § Zhejiang Provincial Key Laboratory for Model Organisms, School of Life Sciences, Wenzhou Medical College, Wenzhou, Zhejiang 325035, and; the ‖ Department of Biochemistry and Molecular Biology, College of Life Sciences, Inner Mongolia University, Huhhot, Inner Mongolia 010021, China

**Keywords:** Arrestin, G Protein-coupled Receptors (GPCR), MAP Kinases (MAPKs), Neuropeptide, Signaling

## Abstract

Corazonin, an undecapeptide neurohormone sharing a highly conserved amino acid sequence across Insecta, plays different physiological roles in the regulation of heart contraction rates, silk spinning rates, the induction of dark color and morphometric phase changes, and ecdysis. Corazonin receptors have been identified in *Drosophila melanogaster, Manduca sexta*, and *Musca domestica*. However, detailed information on the signaling and major physiological functions of corazonin and its receptor is largely unknown. In the current study, using both the mammalian cell line HEK293 and insect cell lines BmN and Sf21, we paired the *Bombyx* corazonin neuropeptide as a specific endogenous ligand for the *Bombyx* neuropeptide G protein-coupled receptor A21 (BNGR-A21), and we therefore designated this receptor as BmCrzR. Further characterization indicated that synthetic BmCrz demonstrated a high affinity for and activated BmCrzR, resulting in intracellular cAMP accumulation, Ca^2+^ mobilization, and ERK1/2 phosphorylation via the G_q_- and G_s_-coupled signaling pathways. The direct interaction of BmCrzR with BmCrz was confirmed by a rhodamine-labeled BmCrz peptide. Moreover, experiments with double-stranded RNA and synthetic peptide injection suggested a possible role of BmCrz/BmCrzR in the regulation of larval growth and spinning rate. Our present results provide the first in-depth information on BmCrzR-mediated signaling for further elucidation of the BmCrz/BmCrzR system in the regulation of fundamental physiological processes.

## Teaser

**Background:** The corazonin system is of importance for insects to control multiple physiological processes.

**Results:** *Bombyx* BNGR-A21 was activated in signaling and internalization by direct interaction with corazonin.

**Conclusion:** *Bombyx* corazonin was a specific ligand for dual G_q_- and G_s_-coupled BmCrzR.

**Significance:** Our findings will lead to a better understanding of the corazonin system in regulation of fundamental physiological processes.

## Introduction

Corazonin (Crz)^3^ is an undecapeptide and is a member of the neurohormone family that is ubiquitously present in arthropods with the exception of beetles, an aphid, and a spider mite (1, 2). Crz was initially purified from the corpora cardiaca of the cockroach *Periplaneta americana* with a functional ability to accelerate the heartbeat (3). The structure of corazonin is conserved and occurs as a single isoform in all insects studied thus far. Crz possesses a pyroglutamated N terminus and an amidated C terminus. To date, six modifications of corazonin have been identified. [Arg^7^]Corazonin (with Arg as the seventh residue from the N terminus) is the most universal in insect lineages (4). *Bombyx* [Arg^7^]corazonin has been isolated from the larval brain of the silkworm, *Bombyx mori* (5), and it was found to colocalize with the circadian clock marker proteins Doubletime and Period (6). Studies have also demonstrated that all residues are required for the interaction of the peptide with its receptor, which is consistent with its conserved structure during evolution (7–9).

Corazonin executes diverse functions in various insects. Besides its initial assignment as an accelerator of heartbeat in cockroaches, additional functions ranging from pigment migration in the eye of locusts, melanization of the locust cuticle to initiating the ecdysis, and clock functions in the moth *Manduca sexta* (10, 11). In *Drosophila* corazonin is supposed to regulate various stress responses, metabolism, female fecundity, and probably the downstream principal clock neuron (12–16). It had been reported that injected corazonin reduced the spinning rate, cocoon size, and hemolymph ecdysteroid level in the silkworm, meanwhile prolonging the pupal period (17). However, recent data indicated that RNA interference failed to detect a significant role for this neuropeptide in modulating mosquito heart physiology (18). Besides, [His^7^]corazonin has been shown to play an important role in the regulation of the nutritional and physiological stress response in other insects (14, 15, 19, 20). However, the function of corazonin neuropeptides in insects remains obscure.

The physiological function of corazonin peptides is mediated by the corazonin receptor. The first corazonin receptor (CrzR) was identified from the fruit fly *Drosophila melanogaster* in 2002 (21), followed by the identification of homologous receptors in *M. sexta* (11), *Anopheles gambiae* (22), *Apis mellifera* (23), and *Musca domestica* (24). These receptors all possess the typical features of a seven transmembrane G protein-coupled receptor (GPCR). The transmembrane regions of these receptors are conserved, whereas the N terminus is highly divergent. In *Drosophila*, corazonin has been identified as the endogenous ligand for the G protein-coupled receptor CG10698, and this CrzR is expressed in embryos, larvae, pupae, and adult flies. In *M. sexta*, the corazonin receptor exhibited high sensitivity and selectivity for corazonin in *Xenopus* oocytes and was used as a biosensor to verify the initiation of the ecdysis behavioral sequence in Inka cells by corazonin signaling (11). The CrzR in *A. gambiae* was found to only be activated by its endogenous corazonin, with an EC_50_ value of 4 × 10^−9^ m, resulting in cAMP accumulation (22). In *M. domestica*, tissue-specific RT-PCR revealed a high level of *M. domestica* corazonin receptor expression in the larval salivary glands and a moderate level in the CNS, whereas in adults, the receptor was expressed in both the head and body, suggesting multifunctionality of the Crz signaling system (24).

Recent studies have highlighted the physiological functions of corazonin, such as rhythm control, metabolism, ecdysis, and stress response. However, the detailed information on Crz/CrzR signaling remains to be further elucidated. By genomic data mining and phylogenetic analysis, the *Bombyx* neuropeptide G protein-coupled receptor BNGR-A21 has been identified as a corazonin-like receptor in *B. mori* (25–27). Although adipokinetic hormone (AKH), which mobilize lipids and carbohydrates from the insect fat body, and corazonin receptors are closely related to the gonadotropin-releasing hormone (GnRH) receptors (28–30), our previous data suggested that BNGR-A21 is independent of other members of the AKHR family by comparing the signaling and internalization of *Bombyx* AKHR, AKHR2a, and AKHR2b in the silkworm (31, 32). Therefore, in this study, we report on the cloning of the cDNA encoding the putative BNGR-A21 from the silkworm *B. mori* and its functional expression in the mammalian cell line HEK293 and insect cell lines BmN and Sf21. Our characterization provides an outline for Crz/CrzR signaling and perhaps aids in the interpretation of the signaling in ecdysis, spinning, development, and stress response in *B. mori*.

## EXPERIMENTAL PROCEDURES

### 

#### 

##### Materials

Cell culture media and fetal bovine serum (FBS) were purchased from HyClone (Beijing, China). G418, Lipofectamine 2000, and Opti-MEM® I reduced serum medium were purchased from Invitrogen. SuperFectin^TM^ II was purchased from Pufei Biotech (Shanghai, China). The pEGFP-N1 and pCMV-FLAG vectors were purchased from Clontech Laboratories, Inc. and Sigma, respectively. Monoclonal anti-FLAG® M2 antibody, monoclonal anti-FLAG M2-FITC antibody, horseradish peroxidase (HRP)-conjugated anti-mouse IgG, and Nifedipine were purchased from Sigma. Anti-phospho-ERK1/2 (Thr^202^/Tyr^204^) and anti-ERK1/2 antibodies were purchased from Cell Signaling Technology (Danvers, MA). H89, Go6983, U73122, U0126, cholera toxin (CTX), and pertussis toxin (PTX) were purchased from Tocris Bioscience. The membrane probe DiI (1,1′-dioctadecyl-3,3,3′,3′-tetramethylindocarbocyanine perchlorate), nuclear dye, RIPA lysis buffer, and horseradish peroxidase-conjugated secondary antibody were purchased from Beyotime (Haimen, China). [Arg^7^]Corazonin was synthesized by GL Biochem Ltd. (Shanghai, China).

##### Cloning of the BNGR-A21 cDNA and Construction of the Mammalian and Insect Expression Vectors

To construct the BNGR-A21 plasmid, RT-PCR was performed as described previously (32). To amplify the full-length sequence encoding BNGR-A21, two pairs of primers for the receptor were designed based on the sequence from the GenBank^TM^ AB330442 as described below. The coding sequence of A21 was amplified for the pCMV-FLAG vector using the sense primer 5′-AAGCTTATGGACAACGAAGGCAACAGTAC-3′ and the antisense primer 5′-GGATCCTTATAACAAACTAATGTTCTGTCCAT-3′; an additional pair of primers was also designed for the pEGFP-N1 vector: the sense primer 5′-AAGCTTGCCACCATGGACAACGAAGGCAACAGTAC-3′ and the antisense primer 5′-GGATCCCGTAACAAACTAATGTTCTGTCCATTCG-3′. The corresponding PCR products were cloned into the pCMV-FLAG, pEGFP-N1, pBmIE1-FLAG, and pBmIE1-EGFP expression vectors using the Rapid DNA Ligation Kit (Beyotime, China) and we named these vectors Flag-A21, A21-EGFP, BmFlag-A21, and BmA21-EGFP, respectively. All constructs were sequenced to verify the correct sequences, orientations, and reading frames. Arrestin3-EGFP was generated as previously described (33).

##### Cell Culture and Transfection

The human embryonic kidney cell line (HEK293) was maintained in Dulbecco's modified Eagle's medium (DMEM) supplemented with 10% heat-inactivated fetal bovine serum (HyClone) and 4 mm l-glutamine (Invitrogen). The BNGR-A21 cDNA plasmid constructs were transfected or cotransfected into HEK293, BmN, and Sf21 cells using Lipofectamine 2000 (Invitrogen) and SuperFectin^TM^ II (Pufei, China) according to the manufacturer's instructions. Selection for stable expression was initiated by the addition of G418 (800 μg/ml) 1–2 days after transfection.

##### Flow Cytometry Analysis

Approximately 2 × 10^5^ cells were washed with phosphate-buffered saline (PBS) containing 0.5% BSA (FACS buffer) and incubated with 10 μg/ml of FITC-labeled anti-FLAG M2 monoclonal antibody (Sigma) in a total volume of 100 μl. After incubation for 60 min at 4 °C, the cells were pelleted and washed three times in FACS buffer. The cells were then fixed with 4% paraformaldehyde in FACS buffer and subjected to flow cytometry analysis on a FACScan flow cytometer (Cytomics FC 500 MCL, Beckman Coulter).

##### cAMP Assay and Accumulation Measurement

After seeding in a 48-well plate overnight, HEK293 or Sf21 cells transiently or stably cotransfected with BNGR-A21 and pCRE-Luc were grown to 90–95% confluence, stimulated with the corazonin peptides in DMEM without FBS, and incubated for 4–6 h at 37 °C. Luciferase activity was detected by a firefly luciferase assay kit (KenReal, Shanghai, China). The cAMP concentration was assessed using a commercially available cAMP detection kit (R&D Systems).

##### Intracellular Calcium Measurement

The stable FLAG-A21- and BmFlag-A21-expressing HEK293 and Sf21 cells were washed twice with phosphate-buffered saline and resuspended at 5 × 10^6^ cells/ml in Hanks' balanced salt solution. The cells were detached by a Nonenzymatic Cell Dissociation Solution (M&C Gene Technology, China) or 0.02% EGTA and then loaded with 3 μm Fura 2-AM (Dojindo Laboratories, Japan) for 30 min at 37 °C. Cells were washed twice in Hanks' solution and then resuspended in Hanks' solution at a concentration of 3 × 10^7^ cells/ml. Calcium flux was measured using excitation wavelengths of 340 and 380 nm in a fluorescence spectrometer (Tecan Infinite 200 PRO, Switzerland).

##### ERK1/2 Activation Assay

The HEK293 and Sf21 cells transiently or stably expressing BNGR-A21 were seeded in 24-well plates and starved for 60 min in serum-free medium to reduce background ERK1/2 activation and to eliminate the effects of the change of medium. After stimulation with the agonist, the cells were lysed by RIPA buffer (Beyotime, China). Equal amounts of total cell lysate were size-fractionated by SDS-PAGE (10–12%) and transferred to a PVDF membrane (Millipore). Membranes were blocked in blocking buffer (TBS containing 0.05–0.1% Tween 20 and 5% nonfat dry milk) for 1 h at room temperature and then incubated with rabbit monoclonal anti-phospho-ERK1/2 antibody (Cell Signaling) and anti-rabbit HRP-conjugated secondary antibody (Beyotime, China) according to the manufacturers' protocols. Total ERK1/2 was assessed as a loading control after p-ERK1/2 chemiluminescence detection using an HRP substrate purchased from Cell Signaling (Danvers, MA).

##### Measurement of Cell Surface Receptors by ELISA

HEK293 cells stably transfected with the FLAG-A21 construct were seeded in 24-well dishes coated with poly-l-lysine. The cells were stimulated with the indicated concentrations of the agonist at the indicated times the following day. The medium was aspirated, and the cells were washed once with Tris-buffered saline (TBS). After fixing the cells for 5 min at room temperature with 3.7% formaldehyde in TBS, the cells were washed three times with TBS and then blocked for 60 min with 1% bovine serum albumin/TBS followed by incubation with horseradish peroxidase (HRP)-conjugated goat anti-mouse antibody (1:5000 in 1% BSA/TBS) for 60 min. To each well, 200 μl of HRP substrate (Sigma) was added, and the samples were incubated at 37 °C for 20–30 min. The reactions were stopped by adding an equal volume of 1% SDS, and plates were read at 405 nm in a microplate reader (Tecan, Switzerland).

##### Confocal Microscopy

For receptor surface expression analysis and internalization assays, HEK293 and BmN cells transiently or stably expressing receptor-EGFP were seeded onto glass coverslips coated with 0.1 mg/ml of poly-l-lysine and allowed to attach overnight under normal growth conditions. After 24 h, cells were stained with the membrane probe DiI (Beyotime, China) at 37 °C for 5–10 min, fixed with 4% paraformaldehyde for 15 min, and finally incubated with DAPI for 10 min. For the internalization assay, cells expressing A21-EGFP were treated with different concentrations of corazonin for different incubation times at 37 °C. For the β-arrestin and kurtz translocation assays, cells were cotransfected with the corresponding FLAG-A21 receptor constructs and β-arrestin1-EGFP, β-arrestin2-EGFP, or Bmkurtz-EGFP. After transfection, the cells were seeded onto glass coverslips, allowed to recover for 24–36 h in a 6-well plate, and incubated with 1 ml of DMEM without FBS, and if necessary, the corresponding ligands were added to stimulate the receptors for 5–15 min. The cells were visualized by fluorescence microscopy on a Zeiss LSM 510 laser scanning confocal microscope attached to a Zeiss Axiovert 200 microscope using a Zeiss Plan-Apochromat ×63, 1.40 NA oil immersion lens.

##### Ligand Competition Binding Assay

HEK293 cells stably expressing the FLAG-A21 construct were plated onto poly-d-lysine-coated 96-well plates 1 day before the experiments. Cells were washed once with PBS, and 50 μl of PBS with 0.2% BSA was added to each well. Samples of labeled and unlabeled peptides were made in ligand buffer at a concentration four times the final concentration specified. An amount of 25 μl of labeled ligand and 25 μl of buffer or unlabeled ligand was added to each well. Cells were incubated at room temperature for 90 min and transferred onto ice. Cells were then washed three times with 100 μl of ice-cold PBS with 0.1% BSA as previously described (34). Binding was determined by measuring the fluorescence intensity with a Tecan microplate reader. Binding is presented as the percentage of total binding. The binding displacement curves were analyzed by GraphPad Prism.

##### Quantitative Real-time PCR

Quantitative RT-PCR was performed as described previously with slight modifications (21). Total RNA was extracted from tissue samples as detailed in the manufacturer's protocol (Qiagen). Reverse transcription was completed using the PrimeScript First Strand cDNA Synthesis Kit (TaKaRa, China). The cDNA from the samples was quantified on a real-time PCR machine (CFX-Touch, Bio-Rad) using SYBR Premix Ex Taq (TaKaRa, China). BNGR-A21 used the sense primer 5′-TTCTGAATCCAGACAAAAACCAA-3′ and the antisense primer 5′-ATGATGTGTTGTTATCACCACGA-3′; actin used the sense primer 5′-CGTTCGTGATATCAAGGAGAAGCT-3′ and the antisense primer 5′-TCCATACCCAAGAACGAGGGTTG-3′. The possibility of genomic DNA contamination was excluded by DNase treatment. Differential expression of the cell lines was compared using the ΔΔ*C_T_* method.

##### Peptide Synthesis

The corazonin peptides were prepared by solid-phase synthesis using the *N*-(9-fluorenyl)methoxycarbonyl strategy on a 430A peptide synthesizer (Applied Biosystems, Foster City, CA) and a 9050 Pepsynthesizer Plus (Perceptive Biosystems, Cambridge, MA). Crude peptides were purified by preparative reverse-phase high-performance liquid chromatography using a Dynamax-300 Å C18 25 cm × 21.4-mm inner diameter column with a flow rate of 9 ml/min and two solvent systems of 0.1% TFA/H_2_O and 0.1% TFA/acetonitrile. Fractions containing the appropriate peptide were pooled together and lyophilized. The purity of the final product was assessed by analytical reverse-phase high-performance liquid chromatography, capillary electrophoresis, and matrix-assisted laser desorption/ionization time-of-flight mass spectrometry.

##### Silkworm Rearing and Bioassay

*B. mori* P50 were reared at 27 ± 3 °C and with a light:dark regime of 12 h:12 h on fresh mulberry leaves. Fourth instar larvae of similar size were divided into different treatment groups in the experiment. These larvae began molting on day 5 of the fourth instar and began spinning on day 7 of the fifth instar. Larvae were immobilized on ice before injection. *B. mori* CrzR dsRNA was generated *in vitro* by the MEGAscript® T7 Kit from Invitrogen according to the manufacturer's instructions using the sense primer 5′-TAATACGACTCACTATAGGGCTTCATTGGCAATGTGGCA-3′ and the antisense primer 5′-TAATACGACTCACTATAGGGGATTCTCGGTCACTATGCTGGT-3′. Corazonin and CrzR dsRNA were dissolved in PBS and diethyl pyrocarbonate/H_2_O, respectively, and injected into fourth and fifth instar larvae using a 10-μl microsyringe through the abdominal segments without injury of the silk glands. PBS without corazonin and pXef dsRNA (from MEGAscript® T7 Kit) were injected as the control. The weight of the larvae was recorded at an identical time each day. Bombycis and remnant mulberry leaves were weighed when completely dry. The cocoon layer and cocoon with pupa were weighed at the end of spinning.

##### Data Analysis

All results are expressed as the mean ± S.E. Data were analyzed using nonlinear curve fitting (GraphPad Prism version 5.0) to obtain EC_50_ values. Statistical significance was determined using Student's *t* test. Probability values that were less than or equal to 0.05 were considered significant.

## RESULTS

### 

#### 

##### Cloning and Functional Expression of the BNGR-A21 Receptor in HEK293 and BmN Cell

**FIGURE 1.**
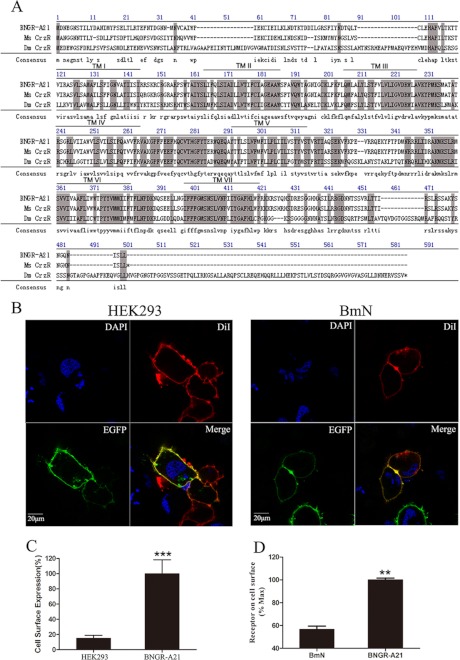
**Protein sequence alignment and expression of BNGR-A21.** *A*, protein sequence alignment of BNGR-A21 with the corazonin receptor from *M. sexta* (MsCrzR accession number AY369029); *D. melanogaster* (DmCrzR accession number AF373862). The seven transmembrane α-helices are indicated by TMI-TMVII. Amino acid residues that are common to these three receptors are *highlighted. B*, HEK293 and BmN cells expressing A21-EGFP fusion protein were stained with a membrane plasma probe DiI and a nuclei probe (*DAPI*). The cell surface expression of FLAG-A21 in HEK293 cells was analyzed by FACS (*C*) and in BmN cells by ELISA (*D*). Receptor cell surface expression was calculated by the value of mean fluorescence intensity (*MFI*), all data are shown as the MFI ratio calculated by dividing the MFI value of cells stained with anti-FLAG mAb by the MFI value for the same cells stained with isotype-matched control Ig. Data are expressed as the mean ± S.D. Data were analyzed by using the Student's *t* test (*, *p* < 0.05; **, *p* < 0.01; ***, *p* < 0.001).

The *Bombyx* neuropeptide receptor BNGR-A21 has been proposed to have a close neighbor-joining phylogenetic tree with the *Drosophila* corazonin receptor by genomic and phylogenetic analysis (26). The full-length cDNA sequence encoding BNGR-A21 (GenBank accession number AB330442.1) was obtained by RT-PCR from the silk gland of silkworm larvae. As shown in Fig. 1*A*, the predicted open reading frame of BNGR-A21 encoding a protein with 437 amino acids contains a typical structure of seven putative transmembrane domains and shows 37.2, 36.7, 46.1, and 81.3% identity to *D. melanogaster*, *A. gambiae*, *A. mellifera*, and *M. sexta* corazonin receptors, respectively. To further assess its functional activity as a transmembrane receptor, BNGR-A21 with an N-terminal FLAG tag and BNGR-A21 with the enhanced green fluorescent protein (EGFP) fused to the C terminus were constructed and stably expressed in human embryonic kidney 293 (HEK293) cells and insect *Spodoptera frugiperda* (Sf21) or *B. mori* (BmN) cells. Significant cell surface expression was detected by FACS and ELISA analysis (Fig. 1*C*) and observed under fluorescence microscopy (Fig. 1*B*), suggesting that the N-terminal FLAG tag and the C-terminal GFP tag did not affect BNGR-A21 expression and orientation in the cell membrane in HEK293 and Sf21 cells.

##### Activation of the BNGR-A21 Receptor by the BmCrz Peptide Dually Couples to the G_q_ and G_s_ Signaling Pathways

**FIGURE 2.**
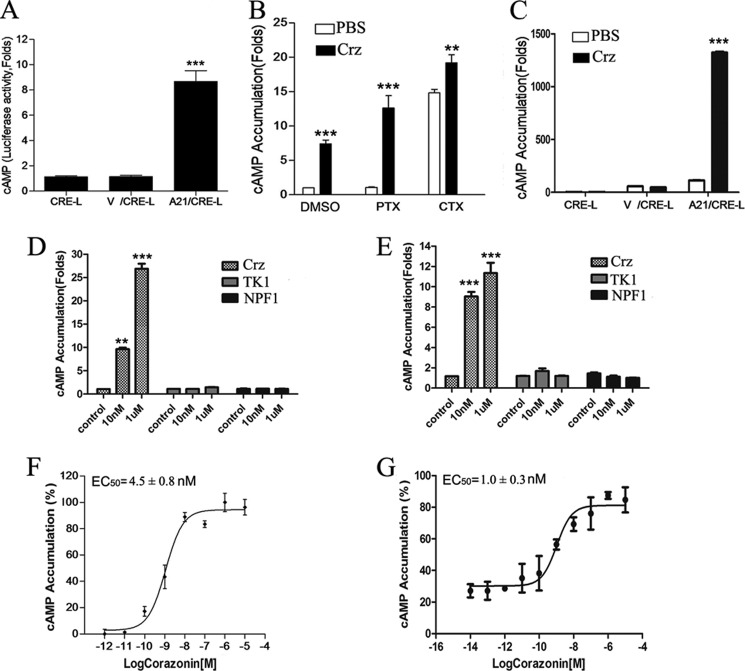
**Corazonin-mediated cAMP formation in BNGR-A21-expressing cells.** *A*, cAMP accumulation in HEK293 cells transiently co-transfected CRE-Luc (*CRE-L*) and BNGR-A21 with or without vehicle vector (*V*) was determined in response to corazonin treatment (1 μm). *B*, effects of PTX or CTX on corazonin-mediated stimulation of cAMP accumulation. HEK293 cells expressing BNGR-A21 were pre-treated with PTX (100 ng/ml) or CTX (300 ng/ml) overnight prior to incubation with coraoznin (1 μm) for 4 h. *C*, cAMP accumulation in Sf21 cells transiently co-transfected with CRE-Luc and FLAG-A21 was determined in response to DMEM (control) and corazonin treatments (1 μm). Effects of tachykinin-1 and neuropeptide F-1 on BNGR-A21-mediated stimulation of cAMP accumulation in HEK293 (*D*) and Sf21 (*E*) cells. cAMP accumulation was assayed in response to different doses of corazonin in HEK293 (*F*) and Sf21 (*G*) cells transiently co-transfected FLAG-A21/CRE-Luc. All data are shown as mean ± S.E. from at least three independent experiments. Data are expressed as the mean ± S.E. Data were analyzed by using the Student's *t* test (*, *p* < 0.05; **, *p* < 0.01; ***, *p* < 0.001).

Previous studies have shown that the corazonin receptor was only activated by its endogenous corazonin, leading to cAMP accumulation, in *A. gambiae* (22), however, the detailed signaling pathways remain to be elucidated. To examine BNGR-A21-mediated G protein coupling and signaling, HEK293 and Sf21 cells were stably cotransfected with BNGR-A21 and a reporter gene system consisting of the firefly luciferase coding region under control of a minimal promoter containing cAMP-response elements. As shown in Fig. 2, *F* and *G*, upon stimulation with corazonin, BNGR-A21 was activated to induce significant accumulation of intracellular cAMP in both HEK293 and Sf21 cells with EC_50_ values of 4.5 ± 0.8 and 1 ± 0.3 nm, respectively, whereas *Bombyx* tachykinin-1 (TK1) and neuropeptide F-1 (NPF1) were unable to activate BNGR-A21 (Fig. 2, *D* and *E*). As a control, no change in the CRE-driven luciferase activity was detected in parental HEK293 and Sf21 cells. Pretreatment with 100 ng/ml of PTX, an inhibitor of Gα_i_ protein, exhibited no effect on cAMP generation in BNGR-A21-expressing cells stimulated by corazonin, whereas stimulation with CTX, which constitutively activates Gα_s_ subunits, led to a remarkable increase in the cellular levels of cAMP (Fig. 2, *A–C*), suggesting that G_s_ was likely involved in the BNGR-A21-mediated signaling in HEK293 and Sf21 cells.

**FIGURE 3.**
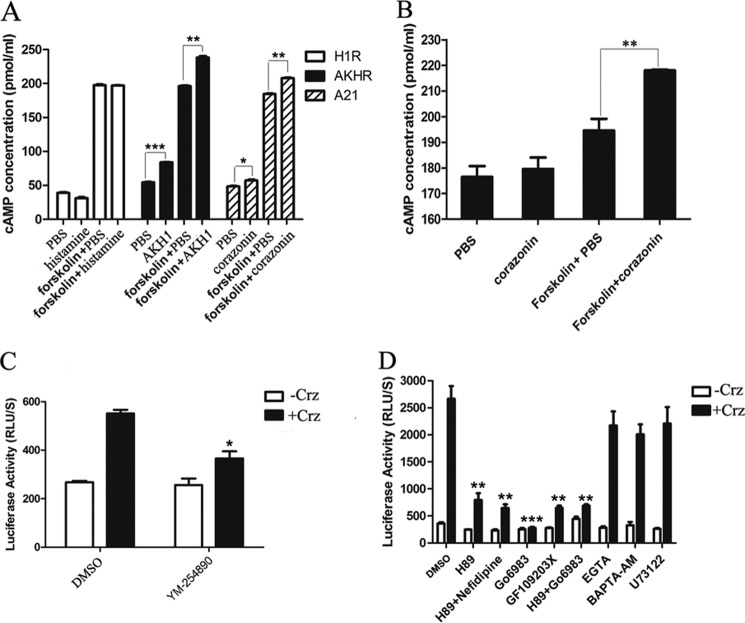
**Corazonin-mediated activation of adenylate cyclase through BNGR-A21.** cAMP accumulation in HEK293 (*A*) and Sf21 (*B*) cells stimulated with DMEM, corazonin (1 μm), DMEM with forskolin (10 μm), and corazonin with forskolin (10 μm) for 10 min was detected by cAMP detection using the ELISA kit. HIR and AKHR were used as controls for G_q_ and G_s_ receptors, respectively. *C* and *D*, effects of different inhibitors on CRE-driven luciferase activity. HEK293 cells were transiently co-transfected with FLAG-A21 and CRE-Luc, and pre-treated with G_q_ inhibitor (YM-254890, 1 μm) (*C*) and PKA (H89, 10 μm), PKC (Go6983,10 μm, and GF109203X, 10 μm), l-calcium channel (nefidipine, 10 μm), calcium chelators (EGTA, 5 mm, and *BAPTA-*AM, 50 μm), and phospholipase C (*PLC*) (U73122, 10 μm) inhibitors (*D*) for 2 h prior to incubation with coraoznin (1 μm) for 4 h. The cAMP concentration was assessed by using a commercially available kit (R&D Systems). Data were analyzed by using the Student's *t* test (*, *p* < 0.05; **, *p* < 0.01; ***, *p* < 0.001). All pictures and data shown are representative of at least three independent experiments.

To confirm whether corazonin can bind to BNGR-A21 and trigger a second messenger cAMP signaling pathway, a direct cAMP assay by ELISA was used in this study. As shown in Fig. 3, *A* and *B*, BmCrz-activated BNGR-A21 triggered a significant increase in intracellular cAMP formation compared with the G_q_-coupled human histamine H1 receptor (H1R), and cotreatment of BmCrz with forskolin induced a significant cAMP accumulation compared with treatment with only forskolin in both HEK293 and Sf21 cells. Moreover, BmCrz-induced luciferase activity was significantly inhibited by treatment with the G_q_ inhibitor YM-254890, and PKA inhibitor H89, PKC inhibitor Go6983, calcium chelators (EGTA and BAPTA-AM), and PLC inhibitor U73122 (Fig. 3, *C* and *D*), indicating that both G_q_ and G_s_ are likely involved in BNGR-A21-mediated through PKA, PKC, Ca^2+^, and phospholipase C activation of CRE-driven reporter transcription.

**FIGURE 4.**
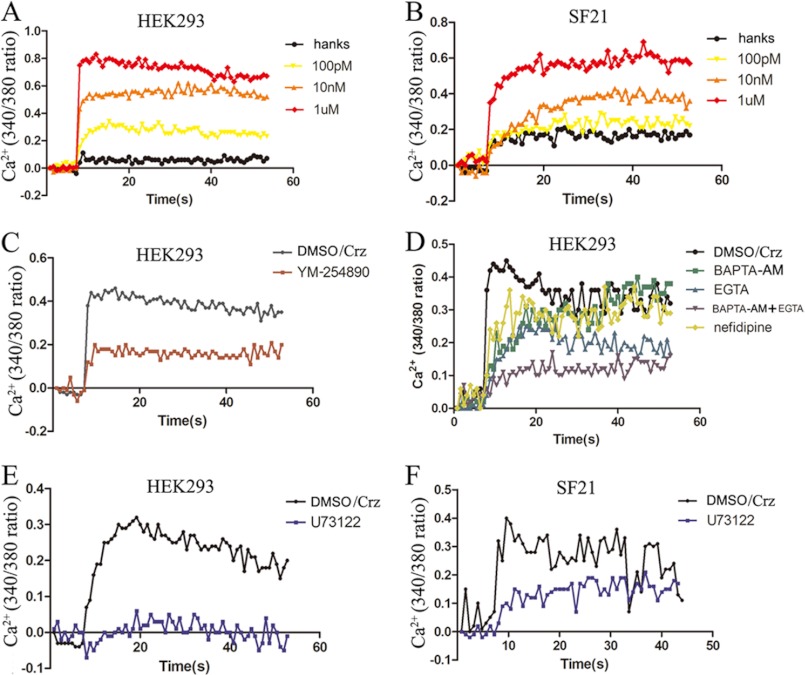
**Intracellular Ca^2+^ influx.** HEK293 (*A*) and Sf21 (*B*) cells, respectively, transfected with FLAG-A21 and BmFlag-A21 were measured in response to different concentrations of corazonin peptide using the fluorescent Ca^2+^ indicator fura-2. *C*, effect of pretreatment of the G_q_ inhibitor on corazonin (1 μm)-mediated Ca^2+^ influx in HEK293 cells. *D*, effects of pretreatment of calcium chelators (EGTA, 5 mm, and BAPTA-AM, 50 μm) and l-calcium channel (nefidipine, 10 μm) inhibitors on corazonin-mediated Ca^2+^ influx in HEK293 cells. Effect of pretreatment of PLC inhibitor (U73122, 10 μm) on corazonin-mediated Ca^2+^ influx in HEK293 (*E*) and Sf21 (*F*) cells. The figures are representative of more than three independent experiments.

We then examined the effects of corazonin peptides on the intracellular Ca^2+^ change in the A21-expressing cells using the calcium probe fura-2. As indicated in Fig. 4, *A* and *B*, corazonin peptides elicited a rapid increase of Ca^2+^ in the FLAG-A21-expressing cells in a dose-dependent manner in both HEK293 and Sf21 cells. Preincubation with 1 μm G_q_ inhibitor YM-254890 led to a significant decrease in the intracellular Ca^2+^ mobilization (Fig. 4*C*). BNGR-A21-mediated intracellular Ca^2+^ mobilization was found to be sensitive to the PLC inhibitor U73122, calcium chelators EGTA and BAPTA-AM, and l-type calcium channel inhibitor nifedipine (Fig. 4, *D--F*). Taken together, these results suggest that the BNGR-A21 receptor was specifically activated by the BmCrz peptide by primarily coupling to the G_q_ signaling pathway and also dually coupling to the G_s_ signaling cascade, leading to intracellular Ca^2+^ mobilization and cAMP accumulation. Therefore, we suggest designating BNGR-A21 as the *Bombyx* corazonin receptor (BmCrzR).

##### A Rhodamine Red-labeled BmCrz Directly Binds to and Activates BNGR-A21

**FIGURE 5.**
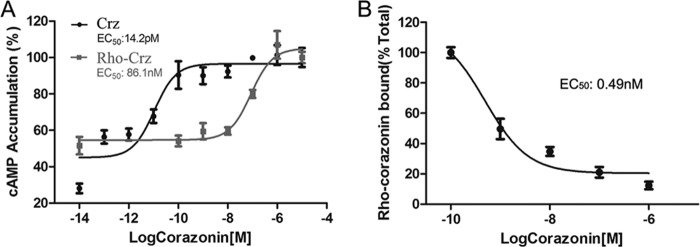
**Direct interaction of BNGR-A21 with corazonin.** *A*, rhodamine red-labeled [Lys^4^]corazonin activity was assayed using the CRE-driven luciferase system. *B*, binding of Rho-[Lys^4^]BmCrz to BNGR-A21 expressed in HEK293 cells was measured in the presence of different concentrations of unlabeled peptides or in the absence of unlabeled peptides (total binding). The extent of binding was determined by fluorescence intensity and is presented as a percentage of the total binding. The results represent the mean ± S.D. (*n* = 3). All images and data are representative of at least three independent experiments.

To confirm the direct interaction of BmCrz peptides with BNGR-A21 receptors, we designed and synthesized a fluorescent BmCrz analog conjugated with rhodamine red. Although corazonin has been shown to be highly conserved in structure, sequence alignment demonstrates the existence of a few mutants including Q1E, Q4T, and R7H. Therefore, we synthesized the Q4K peptide and conjugated it with rhodamine red at the Lys residue (Rho-[Lys^4^]BmCrz). Functional assays indicated that the Rho-[Lys^4^]BmCrz peptide could activate BmCrzR with an EC_50_ value of 86.1 nm (Fig. 5*A*). Using a displacement analysis method, Rho-[Lys^4^]BmCrz was found to compete with unlabeled BmCrz, with an IC_50_ value of 0.49 nm (Fig. 5*B*), suggesting that BmCrz directly binds and activates BmCrzR.

##### Corazonin Activates the ERK1/2 Signaling Pathway via BmCrzR

**FIGURE 6.**
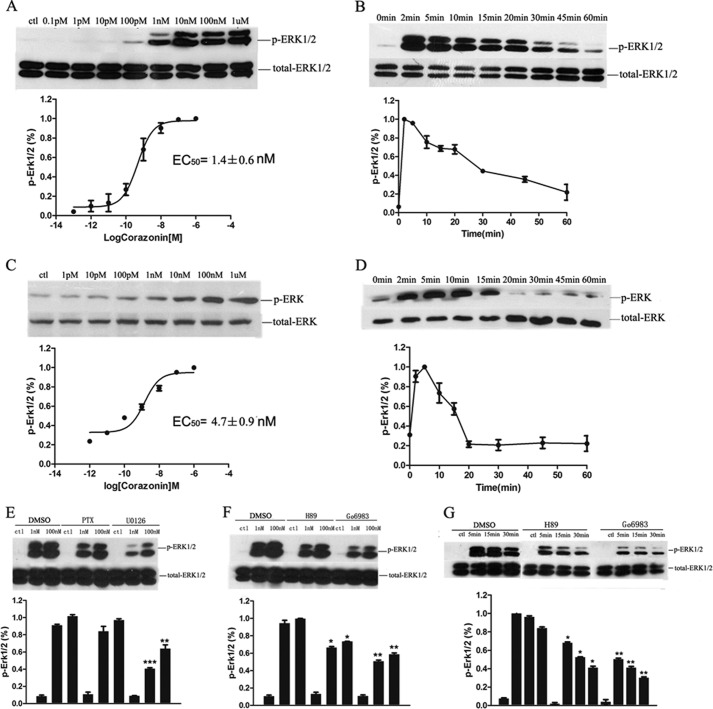
**Effects of inhibitors PKA, PKC, and MEK on corazonin-mediated ERK1/2 activation in BNGR-A21-expressing cells.** Concentration dependence (*A*) and time course (*B*) of corazonin-stimulated phosphorylation of ERK1/2 in stable BNGR-A21-expressing HEK293 cells. Concentration dependence (*C*) and time course (*D*) of corazonin-stimulated phosphorylation of ERK1/2 in Sf21 cells transfected with BNGR-A21. *E*, dose-dependent effects of pertussis toxin (*PTX*, 100 ng/ml) and MEK inhibitor U0126 (1 μm) on BNGR-A21 mediated activation of ERK1/2. Dose-dependent (*F*) and time course (*G*) of PKA inhibitor H89 (10 μm) and PKC inhibitor Go6983 (10 μm) on BNGR-A21-mediated activation of ERK1/2. The cells were pretreated with or without (control) inhibitors for 1 h and then stimulated with corazonin (1 nm, 5 min). The p-ERK was normalized to a loading control (*total-ERK*). The data shown are representative of at least three independent experiments. Statistical analysis was performed by a two-tailed Student's *t* test (**, *p* < 0.01; ***, *p* < 0.001, *versus* counterpart control).

It has been well established that the activated GPCRs signal the ERK1/2 signaling pathway, which functions in transcriptional regulation that is involved in control of diverse processes ranging from proliferation and differentiation to apoptosis and can be used to assess the functional outcome of receptor stimulation (35, 36). We next sought to assess corazonin-mediated activation of ERK1/2 in both HEK293 and Sf21 cell lines stably expressing BmCrzR using a phosphospecific antibody known to bind only to the phosphorylated (Thr^202^ and Tyr^204^ of ERK1 and Thr^185^ and Tyr^187^ of ERK2) forms of these kinases (37). As indicated in Fig. 6, *A–D*, corazonin treatment did not provoke any appreciable effects on ERK1/2 in the parental or transiently mock-transfected HEK293 and Sf21 cells, whereas in the BmCrzR-stably transfected HEK293 and Sf21 cells, stimulation with corazonin elicited transient activation kinetics of ERK1/2 with maximal phosphorylation evident at 2 min in HEK293 cells and 5 min in Sf21 cells, which returned to nearly basal levels by 15 min. A dose-dependent curve indicated that the response to corazonin had an EC_50_ value of 1.4 ± 0.6 nm in HEK293 cells and 4.7 ± 0.9 nm in Sf21 cells for BmCrzR (Fig. 6). In addition, different inhibitors were used to elucidate the signaling pathways involved in the activation of ERK1/2 in HEK293 cells stably transfected with BmCrzR. We demonstrated that the BmCrzR-mediated activation of ERK1/2 was significantly blocked by the PKA inhibitor H89, PKC inhibitor Go6983, and MEK inhibitor U0126 in a dose- and time-dependent manner (Fig. 6, *E-G*) but not by the EGFR tyrosine kinase inhibitor AG1478, PI3K inhibitor wortmannin, and Src inhibitor PP2, confirming that BmCrz is the major agonist for BmCrzR.

##### Corazonin-activated BmCrzR Undergoes Rapid Internalization

**FIGURE 7.**
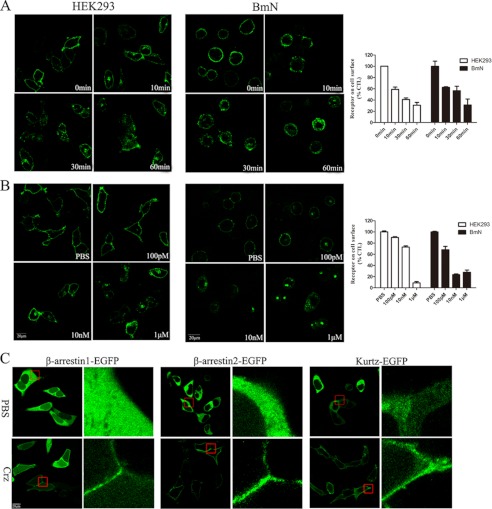
**Internalization of BNGR-A21-expressing cells.** *A*, time course of A21-EGFP internalization induced by corazonin in HEK293 and BmN cells were determined by confocal microscopy and ELISA. *B*, HEK293 cells and BmN cells transfected with A21-EGFP were activated by the indicated concentrations of corazonin for 60 min and detected by confocal microscopy and ELISA. *C*, HEK293 cells transiently co-transfected with arrestin2-EGFP, arrestin3-EGFP, and kurtz-EGPF with FLAG-A21, respectively. β-arrestin1, β-arrestin2, and kurtz resided in the cytosol prior to corazonin stimulation (*PBS*) and was translocated to bind receptors in the membrane in response to treatment with 1 μm corazonin for 5 min. *Error bars* represent S.E. for three replicates. All of experiments were examined by fluorescence microscopy as described under “Experimental Procedure.” All panels are representative of at least three independent experiments.

Agonist-induced internalization is a well characterized phenomenon for most GPCRs that is believed to contribute to the regulation of the strength and duration of receptor-mediated cell signaling (38). To visualize the internalization and trafficking of BmCrzR, we constructed vectors to express a fusion protein of BmCrzR with EGFP at the C terminus, and functional assays demonstrated that BmCrzR-EGFP functioned normally as wild-type BmCrzR in intracellular Ca^2+^ mobilization and CRE-driven luciferase activity (data not shown). Observation with confocal microscopy revealed that the fluorescence of BmCrzR-EGFP was primarily localized in the plasma membrane and was dramatically and rapidly internalized into the cytoplasm in response to the BmCrz peptide in HEK293 and BmN cells (Fig. 7, *A-D*). The internalization of BmCrzR-EGFP was detectable 5 min after agonist stimulation and reached a maximum within 60 min. The internalized BmCrzRs were largely clustered in the perinuclear region at 30 min (Fig. 7, *B-D*). The quantitative ELISA data are highly consistent with our observation by confocal microscopy. Moreover, we cloned kurtz, a novel nonvisual arrestin in insects, from *B. mori* and constructed vectors to express kurtz and arrestins fused with EGFP at the C terminus. HEK293 cells were cotransfected with BmCrzR and arrestin-EGFP or kurtz-EGFP and were examined by confocal microscopy after exposure to the agonist. As shown in Fig. 7, *E* and *F*, in the presence of the BmCrz peptide, β-arrestin1-EGFP, β-arrestin2-EGFP, and kurtz-EGFP showed the same ability to be recruited to the plasma membrane, although we observed that β-arrestin2-EGFP and kurtz-EGFP, but not β-arrestin1-EGFP, remained associated with receptor throughout internalization (data not shown). Our data on receptor internalization confirm that BmCrz is the major agonist for BmCrzR.

##### Examination of in Vivo Expression and Physiological Roles of BmCrzR

**FIGURE 8.**
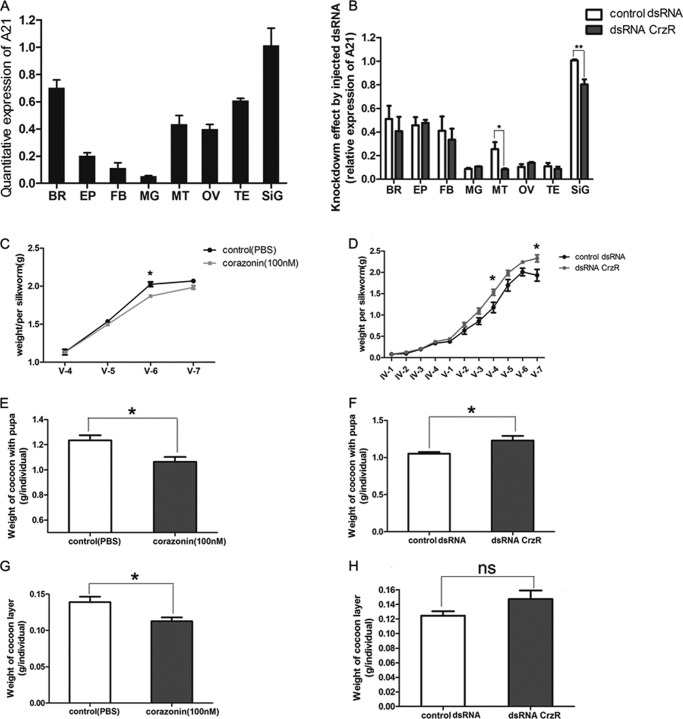
**BNGR-A21 expression in tissues and effects of exogenous corazonin and dsRNA-mediated expression knock-down on silk spinning and growth.** *A*, BNGR-A21 expression in tissues of fifth larval instar. *BR*, brain; *EP*, epidermis; *FB*, fat body; *MG*, midgut; *MT*, Malpighian tubule; *OV*, ovary; *TE*, testis; *SiG*, Poster silk gland. Transcript levels of the BNGR-A21 mRNA were normalized to that of β-actin within each sample. *B*, effect of dsRNA injection on BNGR-A21 expression in different tissues. BNGR-A21 dsRNA (10 μg) was injected on the second day of fifth instar larvae. BNGR-A21 gene expression was determined by RT-PCR 36 h after injection. Each cDNA was analyzed in triplicate and *bars* represent the S.D. *C*, effects of [Arg^7^]corazonin injection on silkworm body weight. *D*, effects of dsRNA treatment on silkworm body weight and cocoon layer. Effects of [Arg^7^]corazonin injection on cocoon with pupa (*E*) and cocoon layer (*G*). Effects of dsRNA treatment on cocoon with pupa (*F*) and (*H*) cocoon layer. All panels are representative of at least three independent experiments (*ns*, not significant; *, *p* < 0.05; **, *p* < 0.01; ***, *p* < 0.001).

To examine the tissue-specific expression of BmCrzR, RT-PCR was used to analyze the expression of BmCrzR in silkworm larvae. As shown in Fig. 8*A*, in fifth instar larvae, BmCrzR expression was detectable in most of the tissues of which the silk gland was found as a major expression site, whereas other tissues, including brain, fat body, epidermis, midgut, Malpighian tubule, testis, and ovary, express BmCrzR at a lower level. This finding is in agreement with the observations made by Yamanaka *et al.* (26). To assess the physiological role of BmCrzR in silkworm larvae *in vivo*, RNAi-mediated knockdown of BmCrzR was performed by the injection of dsRNA at the beginning of the fourth and fifth instar. pXef dsRNA that was provided in the MEGAscript® Kit was injected as a control. As shown in Fig. 8*B*, the mRNA level of BmCrzR in the posterior silk gland and Malpighian tubule was significantly suppressed compared with controls. Although all the larvae that were injected with dsRNA pupated and enclosed normally, the body weight of the larvae and the weight of the cocoon layer markedly increased. In contrast, the larvae receiving the injection of the corazonin peptide demonstrated a significant reduction in body weight and cocoon layer (Fig. 8, *C-H*). These results suggest the possible role of BmCrZ and BmCrzR in the negative regulation of silkworm growth and silk production.

## DISCUSSION

The insect neuropeptide corazonin was initially isolated from cockroaches in 1989 and the first CrzR was identified by functional expression from the fruit fly *D. melanogaster* 10 years ago, however, detailed information on the signaling and physiological roles for corazonin and the corazonin receptor remains largely unknown. Recently, the *Bombyx* neuropeptide GPCR A21 (BNGR-A21) was identified as a corazonin-like receptor in *B. mori* by genomic data mining and phylogenetic analysis (25–27). In the present study, we functionally characterized this putative corazonin receptor with synthetic corazonin peptides using the mammalian cell line HEK293 and insect cell lines BmN and Sf21 stably or transiently transfected with BNGR-A21. Our data demonstrated that this corazonin-like receptor was specifically activated by synthetic *Bombyx* corazonin peptides, leading to intracellular Ca^2+^ mobilization, cAMP accumulation, ERK1/2 phosphorylation, and receptor internalization, but not by *Bombyx* tachykinin, NPF1 or AKH (31). We, therefore, suggest that the BNGR-A21 receptor is better designated as the *B. mori* corazonin receptor (BmCrzR).

To date, five insect corazonin receptors have been cloned and characterized (11, 21–24). In *M. sexta*, the corazonin receptor exhibits high sensitivity and selectivity for corazonin in *Xenopus* oocytes (11). The *A. gambiae* corazonin receptor was found to only be activated by its endogenous corazonin, which had an EC_50_ of 4 × 10^−9^ m, resulting in cAMP accumulation (22). In this study, we first established a cAMP-response element-driven reporter gene assay using both HEK293 and Sf21 cells to functionally analyze the activation of BmCrzR. Our data demonstrated that BmCrzR was activated in response to BmCrz peptides, leading to a significant increase in CRE-driven luciferase activity in a PTX-insensitive but PKA inhibitor H89- and G_q_ inhibitor YM-254890-sensitive manner. This result is also confirmed by direct quantitative analysis of intracellular cAMP using ELISA. These results suggested that G_s_ was likely involved in the BmCrzR-mediated signaling cascades in HEK293 and Sf21 cells, although the G_q_/PLC/PKC signaling pathway was shown to partially contribute to the BmCrzR-induced CRE-driven luciferase activity. Previous studies have shown that G_q_-coupled receptors H1R and prostaglandin EP3 receptor activate adenylyl cyclase through the release of the Gβγ subunits from G proteins (39) or via the G_q_/PLC/Ca^2+^ pathway (40, 41), thereby elevating intracellular cAMP levels. In BmCrzR, more experiments are required to further elucidate the exact mechanism of signaling cross-talk between the G_q_-coupled signaling pathway and the PKA/cAMP-response element-binding protein cascade.

We next used a Ca^2+^ probe to examine the intracellular Ca^2+^ mobilization in both mammalian and insect cells. The BmCrz peptide could elicit a rapid increase of intracellular Ca^2+^ in a dose-dependent manner in both HEK293 and Sf21 cells expressing BmCrzR. BmCrz-triggered Ca^2+^ mobilization was significantly blocked by the G_q_ inhibitor YM-254890 and PLC inhibitor U73122 and was also partially impaired by the calcium chelators EGTA and BAPTA-AM, and the l-type calcium channel inhibitor nifedipine. It is likely that BmCrzR predominantly couples to G_q_ to activate PLC, leading to Ca^2+^ mobilization from the endoplasmic reticulum Ca^2+^ pool to the cytoplasm, but BmCrzR can also modulate PLC or the l-type calcium channel by the βγ subunits released from various G-proteins, resulting in Ca^2+^ influx from the extracellular pool (42–44). Moreover, activated BmCrzR was found to effectively signal to the ERK1/2 transduction pathway in both HEK293 and Sf21 cells, and ERK1/2 activation was significantly inhibited by the PKA inhibitor H89, PKC inhibitors Go6983, and MEK inhibitor U0126 in a dose- and time-dependent manner, which is in high agreement with the observation of intracellular cAMP accumulation and Ca^2+^ mobilization. There are many well documented examples of a GPCR activating different signaling cascades by directly interacting with two different G-proteins. For example, the adrenergic α_1B_ receptor preferentially couples to Gα_q_/PLC to mobilize Ca^2+^ from the ER to the cytoplasm but can also interact with Gα_s_/AC to stimulate intracellular cAMP production (45), and conversely, the histamine H2 receptor predominantly couples to the Gα_s_/AC/cAMP/PKA signaling cascade but also stimulates the Gα_q_/PLC/Ca^2+^/PKA signaling pathway (46). Taken together, these results strongly demonstrate that agonist-activated BmCrzR preferentially couples to G_q_ but also dually couples to G_s_, resulting in the activation of the PLC/Ca^2+^/PKC and AC/cAMP/PKA signaling pathways, respectively.

Internalization is one of the predominant mechanisms that control GPCR signaling for ensuring appropriate cellular responses to stimuli. In this study, we constructed a chimeric protein in which EGFP was fused to the C terminus of BmCrzR (BmCrzR-EGFP). BmCrzR-EGFP that was stably expressed in both HEK293 and Sf21 cells exhibited functional activities in intracellular cAMP accumulation and Ca^2+^ mobilization comparable with wild-type BmCrzR. Using confocal microscopy combined with cell surface ELISA, we found that the BmCrz peptide promoted rapid BmCrzR internalization in a dose- and time-dependent manner. G protein-coupled receptor kinase-mediated phosphorylation and arrestin binding are involved in the regulation of GPCR internalization (47). To confirm the internalization of BmCrzR, we cloned kurtz, a novel nonvisual arrestin in insects (48), from *B. mori* and compared the activity of *Bombyx* kurtz with human β-arrestin1 and β-arrestin2 in the regulation of BmCrzR internalization. GPCR internalization has been characterized by employing an arrestin-GFP fusion protein to monitor the translocation of the arrestin from the cytoplasm to the cell membrane (49). The arrestin-GFP recruitment assay was first successfully used to identify endogenous ligands for *Drosophila* orphan GPCRs (50). The *kurtz* gene encoding the only nonvisual arrestin in *Drosophila* contains both amino- and carboxyl-terminal arrestin domains and shares 72% similarity to the mammalian β-arrestin2 and 74% similarity to β-arrestin1 (48). No differences were found between the receptor-kurtz interactions and the receptor-β-arrestin2 associations (51). Our data strongly suggest that *Bombyx* kurtz behaves more similarly to the mammalian β-arrestin2 in the regulation of BmCrzR internalization than to the mammalian β-arrestin1, suggesting that *Bombyx* kurtz can serve as a useful tool for the characterization of insect GPCR internalization and signaling.

Phylogenetic studies have suggested that not only the AKH and corazonin neuropeptides but also the AKH and corazonin receptors are closely related, apparently originating from one ancestral receptor having one ancestral ligand (26, 28, 52). AKH and the G_s_-coupled AKHR system is well known to mobilize carbohydrates and lipids during energy-expensive activities such as long-distance flight (53), whereas no universal function for corazonin and the corazonin receptor has been recognized, although this neuropeptide shows high conservation in structure and the spatial expression pattern across most insect orders and has been shown to regulate seemingly unrelated functions, including the cardioacceleratory activity in *P. americana* (3), induction of the dark color and morphometric phase changes in locusts (54), reduction of the spinning rates of silk in silkworms (17), initiation of the ecdysis behavioral sequence (11), and regulation of the stress response (15). In this study, quantitative RT-PCR demonstrated that BmCrzR expression was detectable in most of the tissues of which the silk gland was found as a major expression site, whereas other tissues, including brain and fat body, demonstrated lower expression levels, suggesting the possible role of BmCrzR in the regulation of silk gland growth and silk production. Our results from exogenous corazonin injection and dsRNA-mediated knockdown of BmCrzR suggested the possible physiological role of *Bombyx* corazonin and its receptor in the negative regulation of growth and silk production. Therefore, further efforts should be focused on the molecular and functional dissection of the physiological roles played by *Bombyx* corazonin and its receptor.

**FIGURE 9.**
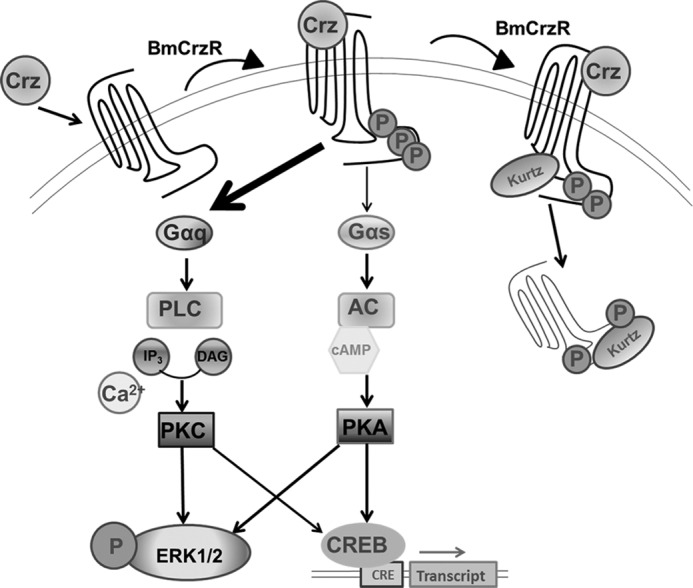
**Schematic diagram of agonist-induced BmCrzR ERK1/2 activation and internalization.** Corazonin binding to BmCrzR activates both G_s_ and G_q_ families of heterotrimeric G proteins, leading to dissociation of G protein subunits. Gβγ, enhanced adenylate cyclase and phospholipase C activity, leading to intracellular cAMP and Ca^2+^ accumulation, which, respectively, activated PKA and PKC, and stimulated phosphorylation of ERK1/2. The binding of corazonin also promoted the recruitment of arrestin or kurtz proteins followed by rapid BmCrzR internalization. *DAG*, diacylglycerol; *CREB*, cAMP-response element-binding protein.

In summary, in the present study, we have identified the *Bombyx* orphan receptor BNGR-A21 as a receptor for the neuropeptide *Bombyx* corazonin. BmCrzR was activated by direct interaction with the corazonin peptide, leading to intracellular Ca^2+^ mobilization via a G_q_-dependent signaling cascade, cAMP accumulation, and ERK1/2 phosphorylation through possibly G_s_-and G_q_-dependent signaling pathways. In response to the corazonin peptide, BmCrzR underwent a rapid internalization via an arrestin-dependent pathway (Fig. 9). Furthermore, functional analysis using exogenous corazonin injection and dsRNA-mediated knockdown of BmCrzR demonstrated a likely role of corazonin and its receptor in the regulation of silkworm growth and silk production. These results will lead to a better understanding of the BmCrz/BmCrzR system in the regulation of fundamental physiological processes.

## Footnotes

3 The abbreviations used are:

Crz: corazonin
CrzR: corazonin receptor
BmCrz: *B. mori* corazonin
GPCR: G-protein-coupled receptor
BNGR: *B. mori* neuropeptide G-protein-coupled receptor
AKHR: adipokinetic hormones receptor
H1R: human histamine H1 receptor
EGFP: enhanced green fluorescent protein
CRE: cAMP-response element
BmCrzR: *B. mori* corazonin receptor
CTX: cholera toxin
PTX: pertussis toxin
dsRNA: double-stranded RNA
BAPTA: 1,2-bis(2-aminophenoxyl)ethane-*N*,*N*,*N*′,*N*′-tetraacetic acid
DiI: 1,1′-dioctadecyl-3,3,3′,3′-tetramethylindocarbocyanine perchlorate.
